# Auditory evoked potentials in a newborn Wistar rat model of hyperbilirubinemia^☆^^☆☆^

**DOI:** 10.1016/j.bjorl.2015.10.008

**Published:** 2015-12-02

**Authors:** Çağıl Gökdoğan, Aydan Genç, Özlem Gülbahar, Ozan Gökdoğan, Ayşe Helvacı, Selin Üstün Bezgin, Leyla Memiş

**Affiliations:** a Gazi University Hospital, Audiology Department, Ankara, Turkey; b Hacettepe University Hospital, Audiology Department, Ankara, Turkey; c Gazi University Hospital, Biochemistry Department, Ankara, Turkey; d Memorial Hospital, ENT Department, Ankara, Turkey; e Gazi University Hospital, Pathology Department, Ankara, Turkey; f Gazi University Hospital, ENT Department, Ankara, Turkey

**Keywords:** Hyperbilirubinemia, Wistar rats, Sensorineural hearing loss, Auditory evoked potentials, Hiperbilirrubinemia, Ratos Wistar, Deficiência auditiva neurosensorial, Potenciais evocados auditivos

## Abstract

**Introduction:**

Hyperbilirubinemia is a common health problem in newborns. Its effects can be different according to the level and duration of the hyperbilirubinemia. The toxic effect of bilirubin on the auditory system can be seen as a sensory neural hearing loss or auditory neuropathy spectrum disorder (ANSD).

**Objective:**

The purpose of our study was to determine the effects of toxic bilirubin level on the auditory system by using Auditory Brainstem Response audiometry.

**Methods:**

Rats are used as animal models due to their low cost and easy attainability. Auditory Brainstem Response was used for auditory assessment. In this study, three groups were established: experimental, control and placebo groups.

**Results:**

In the experimental group, which consists of rats with hyperbilirubinemia, sensory neural hearing loss was found bilaterally in 4 rats (66.67%) and unilaterally in 2 rats (16.67%) and auditory neuropathy spectrum disorder was found unilaterally in 1 rat (8.33%). Auditory Brainstem Response thresholds were significantly elevated compared to control and placebo groups (*p* < 0.05).

**Conclusion:**

Hyperbilirubinemia of newborn rats may result both in sensory neural hearing loss and auditory neuropathy spectrum disorder.

## Resumo

**Introdução:**

A hiperbilirrubinemia é um problema de saúde comum em neonatos. Seus efeitos podem variar, dependendo do nível e da duração da hiperbilirrubinemia. O efeito tóxico da bilirrubina no sistema auditivo pode ser observado na forma de deficiência auditiva sensorioneural ou de distúrbio do espectro da neuropatia auditiva.

**Objetivo:**

A finalidade de nosso estudo foi determinar os efeitos de nível tóxico de bilirrubina no sistema auditivo, com o uso da audiometria da resposta auditiva evocada de tronco cerebral.

**Método:**

Os ratos são empregados como modelos animais graças a seu baixo custo e fácil obtenção. Utilizamos a resposta auditiva evocada de tronco cerebral para avaliação da audição. No estudo, foram estabelecidos três grupos: experimental, controle e placebo.

**Resultados:**

No grupo experimental, constituído de ratos com hiperbilirrubinemia, disacusia auditiva neurosensorial foi diagnosticada bilateralmente em quatro ratos (66,67%), e unilateralmente em dois (16,67%); e distúrbio do espectro da neuropatia auditiva foi observado unilateralmente em um rato (8,33%). Os limiares da resposta evocada de tronco cerebral estavam significantemente elevados, em comparação com os grupos controle e placebo (p < 0,05).

**Conclusão:**

A hiperbilirrubinemia de ratos neonatos pode resultar tanto em disacusia auditiva neurosensorial como em distúrbio do espectro da neuropatia auditiva.

## Introduction

Hyperbilirubinemia is a common health problem of newborns. Newborn hyperbilirubinemia has been accepted as one of the main risk factors in infants’ hearing loss since the 1900s.^1^ Effects of hyperbilirubinemia on hearing function can be different according to the level of hyperbilirubinemia and duration of hyperbilirubinemia.^2^ It is known that a high bilirubin level is a risk factor for sensory neural hearing loss (SNHL) and auditory neuropathy spectrum disorders (ANSD).^3^

SNHL results from pathology in the cochlea, 8th nerve, brain stem or cortex level. Although 50% of SNHL in children is genetic, it can also develop depending on pathologies in the prenatal, natal, or postnatal periods (infections, metabolic disorders etc.).^4^ In postnatal periods hyperbilirubinemia is the most common SNHL cause for newborns, and SNHL prevalence depending on hyperbilirubinemia in newborns and infants has been determined as 12.8%.^5^

ANSD is an issue that has many unknown aspects and it is widely studied. In some cases of ANSD, while only the inner hair cells in the inner ear are affected, in some other cases only the central auditory pathway is affected. Among the ANSD risk factors are hyperbilirubinemia, premature birth, and genetic, perinatal mechanical ventilation. ANSD related to hyperbilirubinemia is seen in 2.7% of newborns with hyperbilirubinemia.^6^

Hyperbilirubinemia may have different pathological effects on the ABR pattern of affected individuals. In newborns with hyperbilirubinemia, the ABR pattern can either go back to normal or become even more pathological after blood bilirubin levels are brought to normal with treatment.^7^ Hyperbilirubinemia in the newborn period has been previously studied with rat model.^8^, ^9^

The purpose of our study is to determine the effects of toxic bilirubin level on the auditory system by using Auditory Brainstem Response audiometry.

## Method

The study has been conducted in the local Experimental Animals Research and Application Center of the Faculty of Medicine. The approval of the ethical board has been taken (number of approval of the ethics committee: G.Ü.ET-12.001).

Three groups have been included in the study, as experiment, placebo and control groups and 6 newborn rats have been used for each group. The number of rats in each group was restricted by the ethics committee. The ear examination of all rats has been performed with endoscopes by an otorhinolaryngologist.

### Experiment group

The experiment group consisted of 6 newborn Wistar Albino male rats with hyperbilirubinemia, with weights between 18 and 20 g (average: 18.33 g). A Rat Hyperbilirubinemia Model has been created for the experiment group.

The method for establishing hyperbilirubinemia was similar to methods previously reported.^9^ Bilirubin (Sigma, St. Louis, MO; B4126) was stored in the dark, and the solution was prepared just before the injection. Bilirubin was thawed in 0.1% M NaOH just as in Hansen et al.^8^ application and stabilized with BSA and diluted with Krebs-Ringer buffer (pH: 7.4). The bilirubin concentration has been determined as 3 mg/mL, and the prepared solution has been preserved at +40 °C, in a shaded place. Each animal in the experimental group received an intraperitoneal injection of bilirubin at 50 mg/kg on the postnatal 7th and 10th days. Prior to the 1st injection and 24 h after the 1st and 2nd bilirubin injections, measurements have been done through Transcutaneous Bilirubinometry (TcB) over the skin, in order to determine the bilirubin level. On the 21st day, electrophysiological hearing tests and TcB were performed after intramuscular anesthesia (50 mL/kg ketamine and 10 mL/kg xylazine). Then euthanasia was performed by drawing intracardiac blood under deep anesthesia. The serum bilirubin level was determined quantitatively from the intracardiac blood drawn and was compared to TcB.

### Placebo group

The placebo group consisted of 6 newborn Wistar Albino male rats with weights between 18 and 20 g (average: 18.3 g). As the placebo solution, BSA and 24 mg/mL Krebs-Ringer buffer (pH: 7.5) have been used. The prepared solution has been preserved in +40 °C, in a shaded place. In order to determine whether it has an effect on the hearing system of rats, the solution which has been prepared without bilirubin has been applied in exactly the same dosage which has been applied to the experiment group. The placebo solution has been given in the same procedure applied on the rats in the experiment group and has been subjected to the same processes.

### Control group

The control group consisted of 6 newborn Wistar Albino male rats with weights between 18 and 20 g (average: 19 g). Contrary to the experiment and placebo groups, the rats in the control group have not been given any injections. However, in order to determine their bilirubin levels, coordinated evaluations have been performed with the time slices determined for the experiment and placebo groups and they have been subjected to the same processes.

### Auditory Brainstem Response (ABR) test

The ABR evaluations of the rats included in the study have been performed with the Bio-Logic Systems Corp.’s Navigator Pro Model (version 2.2.0) device. In the ABR evaluations, 13.00 rate click stimulus, 10 msn analysis time, 1000 sweep in averaging, 100–1500 Hz filtration have been used. Changes in the intensity have been made in accordance with the responses achieved in the 70 dB nHL intensity level recordings. In order to determine the CM existence, the condensation (+) rarefaction (−) polarity change has been applied in the same intensity for both ears. For the threshold scan, as it is advised in the click stimulus, it has been continued with rarefaction polarity.

### Transcutaneous Bilirubin (TcB) measurement

Transcutaneous Bilirubinometry (TcB) has been performed with Minolta/Air – Shields Jaundice Meter (JM, mode 101, Minolte Corero Co, Osaka, Japan). TcB measurement has been performed on the back area of the rats. Prior to the measurement, technical maintenance and calibration of the device has been performed. During the measurements, due to the pressure applied by the probe, an acrylic plaque was made to prevent the rats from being damaged.

### Serum Total Bilirubin (STB) measurement

Serum Total Bilirubin levels have been analyzed using readymade kits (Roche) in auto analyzer (Roche/Integra-800) systems. The total bilirubin measurement principle of this kit is the Diazo method based on the measurement of the amount of azobilirubin, which is the result of the reaction of bilirubin and diazotized sulfanilic acid.^10^

### Statistical evaluation

Statistical analysis has been realized in SPSS for Windows Version 16.0 packaged software. The TcB results used to determine the bilirubin levels in the groups have been compared with the STBL, ABR findings for the hearing evaluation differences in the groups, hasn’t been normally distributed the Kruskal–Wallis Variance analysis has been applied. The level of significance has been taken as *p* < 0.05. The Mann–Whitney *U* test has been performed to test the significance of pair wise differences using Bonferroni correction to adjust for multiple comparisons (0.05/3 = 0.016). An overall 5% type I error level has been used to infer statistical significance. The correlation coefficient of the relationships between the variables in the groups and statistical significance has been calculated by the Spearman test. An overall 5% type I error level has been used to infer statistical significance.

## Results

### Levels of Transcutaneous Bilirubinometry (TcB) and Serum Total Bilirubin

While the comparison of the groups in terms of the TcB results (Table 1) prior to the bilirubin injection has not been observed to be statistically meaningful (*p* > 0.05), the TcB results performed 24 h after the 1st and 2nd bilirubin injections show that there is a statistically higher level of bilirubin (*p* < 0.05) in the rats in the experiment groups compared to the rats in the placebo and control groups. When the STBL in blood is compared between the groups, it has been observed that it is higher in statistically significant terms in the experiment groups in comparison to the placebo and control groups. It has been observed that there is a statistically significant positive and strong relationship between the TcB value prior to euthanasia and the STBL value in the experiment, placebo and control groups.

**Table 1:** TcB, STB (mg/dL) values and ABR (dBnHL) thresholds of rats.

Rats	Groups	TcB1	TcB2	TcB3	TcB4	STB	ABR right	ABR left
1	Experiment	0.01	0.89	0.72	0.56	0.61	30	40
2		0.01	0.86	0.62	0.4	0.23	30	30
3		0.02	0.92	0.7	0.2	0.15	10	10
4		0.01	0.96	0.69	0.5	0.58	NR	NR
5		0.03	0.72	0.4	0.28	0.23	20	30
6		0.02	0.9	0.74	0.62	0.69	40	50
1	Placebo	0.01	0.03	0.02	0.38	0.46	20	0
2		0.01	0.02	0.04	0.4	0.43	NR	NR
3		0.02	0.05	0.04	0.28	0.33	20	20
4		0.02	0.02	0.05	0.22	0.3	−10	0
5		0.01	0.05	0.03	0.38	0.44	10	10
6		0.01	0.02	0.04	0.3	0.42	10	10
1	Control	0.03	0.03	0.04	0.06	0.08	NR	NR
2		0.01	0.01	0.03	0.15	0.17	20	10
3		0.01	0.01	0.02	0.1	0.17	10	20
4		0.01	0.01	0.02	0.02	0.03	10	20
5		0.02	0.02	0.04	0.09	0.15	10	10
6		0.01	0.01	0.03	0.1	0.17	10	0

TcB1, before bilirubin injection; NR, no response; TcB2 and 3 results performed 24 h after the 1st and 2nd bilirubin injections; TcB4 value prior to euthanasia.

### Auditory Brainstem Responses (ABR)

#### Threshold results

The comparison of ABR between the groups, one way variance analysis has been used. According to the results of the analysis, while right ear ABR threshold average does not show any significant difference between the groups (*p* > 0.05), left ear ABR threshold shows a statistically significant difference between groups (*p* < 0.05). This difference is a result of the fact that the left ear threshold intensity of the rats in the experiment groups is higher compared to the other two groups (*p* < 0.016) (Table 1, Table 2). For the test of the relationship between the STBL values in the study groups and ABR thresholds, Spearman's rho coefficient has been calculated between the variables. According to the results, there is no significant level of relationship between the ABR left and right threshold values with the STBL value in terms of the groups.

**Table 2:** Mean threshold of ABR in right and left ears (dB nHL).

	Group	Mean	SD	Median	Min–max
Right ear	Control	12.00	4.47	10	10–20
ABR threshold	Placebo	10.00	12.25	10	−10–20
	Experiment	26.00	11.40	30	10–40
Left ear	Control	12.00	8.37	10	10–20
ABR threshold	Placebo	8.00	8.37	10	0–20
	Experiment	32.00	14.83	30	10–50

**Figure 1:**
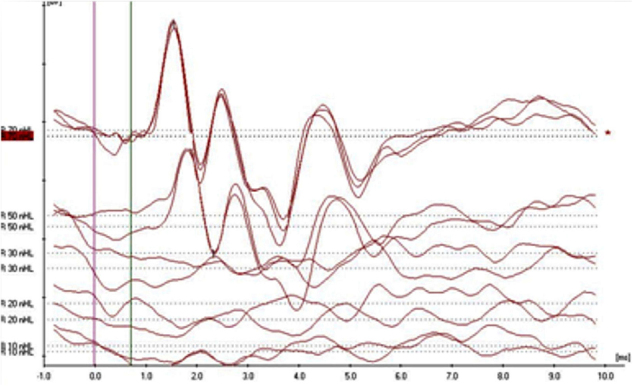
Example for Category 1 (normal ABR pattern).

**Figure 2:**
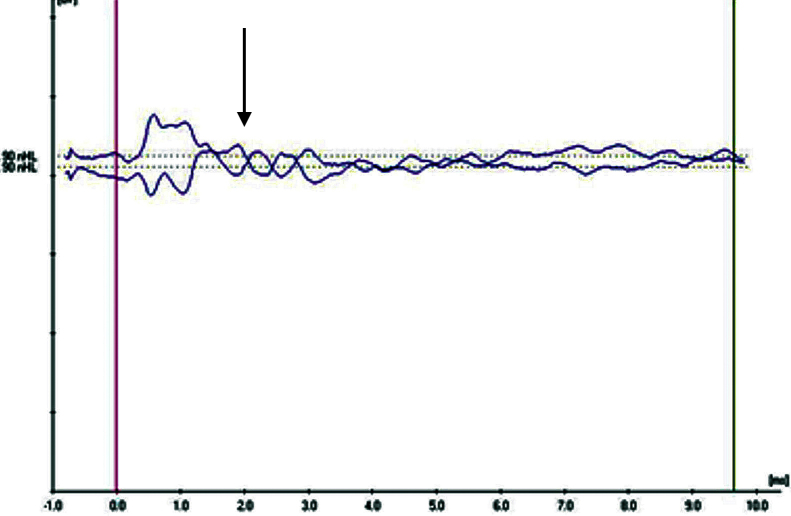
Example for Category 2 (Cochlear Microphonic pattern without ABR threshold).

**Figure 3:**
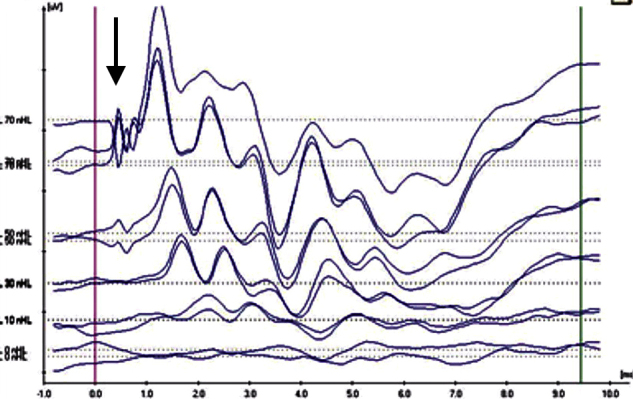
Example for Category 3 (normal ABR with Cochlear Microphonic).

**Figure 4:**
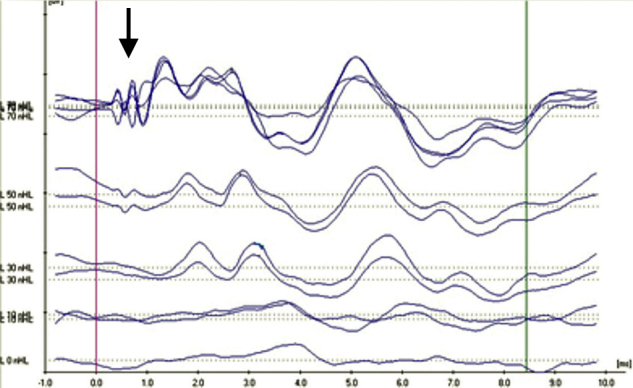
Example for Category 4 (the broken wave morphology including the 2nd wave Cochlear Microphonic).

In the evaluation of the CM existence in the ABR pattern achieved from the groups, four aspects have been noteworthy. CM findings have been categorized by taking these observed aspects as a basis. In Category 1 (Fig. 1), while CM has not been achieved with the normal ABR pattern; in Category 2 (Fig. 2), CM has been observed without the 2nd wave (the ABR threshold was considered to be the lowest intensity at which a definite repeatable response peak II was present). In addition, the category achieved in the 2nd wave with the CM has been evaluated as Category 3 (Fig. 3) and the broken wave morphology including the 2nd wave with the CM has been evaluated as Category 4 (Fig. 4).

## Discussion

Hyperbilirubinemia is an important health problem in newborns. The hearing system is also sensitive to bilirubin toxicity. The damages in the central auditory areas have been defined in both animals and humans with hyperbilirubinemia in anatomical and functional terms. However, the physiological effects and neuromechanism of the damages caused by hyperbilirubinemia in the peripheral auditory organs are still a mystery.

It has been stated in the literature that ANSD may be observed in 1/3–1/2 of the cases with a high level of hyperbilirubinemia.^11^, ^12^ SNHL may also be observed in hyperbilirubinemia (12.8%).^5^

Bilirubin toxicity is closely related to STBL. While a high correlation is observed between level of bilirubin in humans and SNHL, this correlation decreases in ANSD; although in levels higher than 20 mg/dL the possibility of observing ANSD increases.^3^ There are limited publications which define such definite differences and relationships with pathologies in animal models. Due to the difficulties and limitation in forming animal models, it is difficult to clearly determine bilirubin levels and pathologies resulting from this.^13^

In the forming of a rat model with hyperbilirubinemia, bilirubin injection is frequently resorted to, due to the easiness of access and application and achieving absorbed neurotoxicity in the circulatory system in a short time.^13^, ^14^ Gao et al.,^14^ in their study conducted in 2011, have stated that free bilirubin application is efficient in utilization in both hyperbilirubinemia and formation of ANSD. For this reason, intra-abdominal bilirubin injection has been preferred in the formation of rat model with hyperbilirubinemia in this study.

Hai Bo Ye et al.^13^ state that, in cases where a single and low dosage bilirubin injection is done in animal models, the damage is not long lasting and, for this reason, high and repetitive dosages may be more effective in determining the long term effects of hyperbilirubinemia. However, researchers also underline the fact that the highest risk in these manipulations is mortality. In the study, after it was supported with pre-studies, Hansen et al.^8^ model has been used, and bilirubin dosage appropriate for the weight of the newborn rats and which does not cause mortality has been determined.

As Wennberg et al.^15^ state, due to the insufficiency of approach methods in the monitoring of STBL in newborn experiment animals, it is difficult to form a relationship between the bilirubin level and neurotoxicity. In the literature, there are no publications which state STBL in terms of newborn rats. The most important reason for this is that the blood amount in newborn rats is very low (for instance, it is about 1 cc in a rat that is 1 week old) and that the rats die when this blood is taken. This also causes an important problem in ethical terms. However, the TcB method used to determine the bilirubin level in humans, which is non-invasive, can also be used in rats as well as newborns. It is stated in literature that the TcB results are similar to the results of the total serum bilirubin level in blood. Although it is more reliable to observe the STBL in blood, it is stated that TcB can be used as an alternative.^16^ In the study, periodical measurements have been done through transcutaneous bilirubinometry until euthanasia, in order to determine the bilirubin levels of rats. The bilirubin level determined through the measurements prior to euthanasia and STBL's in blood taken through intracardiac methods after euthanasia have been compared and a significant relationship has been determined in line with the literature.

It is stated that the TcB values reach the maximum level 24–48 h after the bilirubin injection in Wistar Albino rats and that these values decrease by time.^8^, ^9^ According to the TcB data achieved in the study, the high level of bilirubin in the experiment group being significantly higher compared to other groups is an important indication that bilirubin toxicity was almost reached. The relative increase of the TcB measurement results prior to euthanasia in all groups may be explained with the variability in the bilirubin values in blood until adulthood.

In newborns, ABR is frequently preferred in the determination of the effect of bilirubin toxicity on the auditory system. ABR, which is used in the evaluation of auditory sensitivity, is preferred in newborns due to its being objective and non-invasive; its reliability being higher compared to other test methods and determination of CM in the identification of ANSD.^17^, ^18^, ^19^

In studies conducted on rats with hyperbilirubinemia, while differences such as latency elongation and decrease in amplitude in ABR have been evaluated, threshold evaluation has also been done along with these variables and it has been observed that there are significant increases in the thresholds of their ABR's.^13^, ^14^, ^19^, ^20^ In the study, while values close to normal data and morphology have been determined in the rats of the control group with the exception of 1 rat, an increase in the threshold values of the ABR findings and deterioration in morphology have been observed in the placebo and experiment groups. In the experiment group, while compatible findings have been achieved in the CM record and ANSD of 1 rat in one ear, it has not been possible to receive ABR response without CM record in the other ear. In other rats, deteriorations in the ABR have been observed in different patterns.

In rats, the anatomical settlement in ABR waves differs from the humans. In humans, while the 1st and 2nd waves originate from the 8th nerve and the 3rd wave originates from the CN, it is known that in rats the 1st and 2nd waves originate from the CN and the 3rd wave originates from SOC.^21^, ^22^, ^23^ The deteriorations observed in the CN in the morphological evaluation conducted in the rats in the experiment group are thought to cause deteriorations in the morphology of the ABR responses in this group as well.^13^, ^14^, ^19^ Shapiro et al.^24^ state that acute bilirubin toxicity especially changes the ABR's significantly in the first 4 h in Gunn Rats.

In the studies conducted on newborns with hyperbilirubinemia, it has been stated that there has been a spontaneous improvement in about 50% without any interventions in the patterns where ABR has not been achieved and CM has been observed.^16^ In addition, the neural damage in the auditory brain stem, which causes ABR change, decreases the synchronization formed by the activation of the auditory neurons. Thus, the membrane potential of the auditory neurons decrease and neural function in the auditory brain stem is corrupted due to hyperbilirubinemia.^13^, ^14^, ^19^, ^20^, ^25^ In our study, due to bilirubin injections being done prior to the formation of blood–brain barriers in the newborn rats, their need for maternal care until adulthood and the risk of mortality becoming higher when they are taken away from their mothers, or during anesthesia, ABR evaluations have not been done immediately after the injections. For this reason, the acute effect of bilirubin toxicity and its follow-up were not evaluated as well.

In the literature, while the difficulty of long term follow-up in animal studies is underlined, it is also noteworthy that the results related to this are not mentioned. In the study, after the 2nd dose injection, we waited for 11 days, until the rats reached adulthood. During this time, it is thought that the potential effects of the hyperbilirubinemia decrease. However, although the effect of hyperbilirubinemia has decreased, as it can be seen from the study as well, due to achieving high intensity ABR thresholds in line with the bilirubin level, this is thought to cause deteriorations in the wave morphology and in being able to achieve ANSD findings and/or ABR.

Podwall et al.^26^ state that, without any risk factor, they have published a pattern that has determined ANSD in the left ear with normal radiological findings, and although bilirubin toxic effect was systemic, unilateral results can also be observed depending on involvement localization.

During the formation of the model, due to the fact that besides the rat type used, the powder bilirubin used was the same in the formation of hyperbilirubinemia, Hansen et al.^8^ model has been used. Due to the fact that the same solution has been used in the placebo group in the literature, a pre-study related to the solution's effects on the auditory system has not been conducted. However, in the evaluation of findings related to the placebo group, similar results have been achieved in the ABR evaluations of some rats compared to the rats in the experiment group.

When it is taken into consideration that the STBL values are not high in the TcB of this group, it gives rise to the thought that the solution is effective on the auditory system as well. This finding, which may be accepted as the weak point of the study, also provides very valuable information in terms of showing understanding of the real effect of hyperbilirubinemia on the auditory system, by studying the effect of the solution to be used in the formation of hyperbilirubinemia in animal models.

As in the study hypothesis, the blood bilirubin levels have been allowed to increase by injecting toxic dosages of bilirubin to newborn rats, and it has been observed that hyperbilirubinemia may cause SNHL and/or ANSD. Besides this, it has also been found noteworthy that the effects of hyperbilirubinemia may appear in different patterns in ABR.

## Conclusion

In our study, by forming rat model for newborns with hyperbilirubinemia through a toxic dose of bilirubin, the effects of level of bilirubin on infants and children under risk, the results of the electrophysiological hearing evaluations and morphological difference have been analyzed.

It has been observed that hyperbilirubinemia can increase the ABR threshold in its long term effects and deteriorate wave morphology. It has been taken under consideration that the deterioration of wave morphology in ABR may be connected to the decrease of synchronization formed by the neural damage in the auditory brainstem and the activation of auditory neurons. It has been observed that hyperbilirubinemia may cause ANSD as well as SNHL and CM can be observed without the ABR response, and that in systemic applications not only bilateral but also unilateral settlements may exist.

## Funding

All financial support of the study was provided by the authors.

## Conflicts of interest

The authors declare no conflicts of interest.
